# Incidence of oxygen desaturation using a high-flow nasal cannula versus a facemask during flexible bronchoscopy in patients at risk of hypoxemia: a randomised controlled trial

**DOI:** 10.1186/s12890-022-02188-4

**Published:** 2022-10-27

**Authors:** Wen Zhang, Jiang-Ling Wang, Shuang Fu, Jia-Ming Zhou, Ye-Jing Zhu, Shu-Nv Cai, Jun Fang, Xin-Zhong Chen, Kang-Jie Xie, Kangjie Xie, Xinzhong Chen

**Affiliations:** 1 Department of Anesthesiology, Research Center for Neuro-Oncology Interaction, The Cancer Hospital of the University of Chinese Academy of Sciences (Zhejiang Cancer Hospital), Institute of Basic Medicine and Cancer (IBMC), Chinese Academy of Sciences, Hangzhou, Zhejiang, China; 2 Department of Anaesthesia, Women’s Hospital, Zhejiang University School of Medicine, Hangzhou, Zhejiang, China; 3 Department of Endoscopy, The Cancer Hospital of the University of Chinese Academy of Sciences (Zhejiang Cancer Hospital), Institute of Basic Medicine and Cancer (IBMC), Chinese Academy of Sciences, Hangzhou, Zhejiang, China; 4 No.1 Banshan East Road, Gongshu District, 310022 Hangzhou, Zhejiang, China; 5 Xueshi Road #1, Shangcheng District, 310006 Hangzhou, Zhejiang, China

**Keywords:** Bronchoscopy, Oxygen desaturation, Deep sedation, High-flow nasal cannula

## Abstract

**Background:**

Patients with obstructive sleep apnoea (OSA), male sex, obesity, older age or hypertension are prone to hypoxemia during flexible bronchoscopy. This study investigated whether using a high-flow nasal cannula (HFNC) could reduce the incidence of oxygen desaturation during bronchoscopy under deep sedation in patients at risk of hypoxemia.

**Methods:**

A total of 176 patients at risk of hypoxemia who underwent flexible bronchoscopy under deep sedation were randomly assigned to two groups: the HFNC group (humidified oxygen was supplied via a high-flow nasal cannula at a rate of 60 L/min and a concentration of 100%, n = 87) and the facemask group (oxygen was supplied via a tight-fitting facemask at a rate of 6 L/min and a concentration of 100%, n = 89).

**Results:**

Oxygen desaturation occurred in 4 (4.6%) patients in the HFNC group and 26 (29.2%) patients in the facemask group (*P* < 0.001). The facemask group required more jaw thrust manoeuvres than the HFNC group (43[48.3%] vs. 5[5.7%], *P* < 0.001). 8 patients (9.0%) in the facemask group and none in the HFNC group required bag-mask ventilation (*P* = 0.012).

**Conclusion:**

The use of an HFNC can reduce the incidence of oxygen desaturation and the requirement for airway intervention in patients at risk of hypoxemia during flexible bronchoscopy under deep sedation.

**Trial registration:**

www.chiCTR.org.cn Identifier: ChiCTR2100044105. Registered 11/03/2021.

## Introduction

Flexible bronchoscopy (FB) is commonly performed under anaesthesia or sedation with a higher acceptability [1]. Hypoxemia can occur in 28.8-56% of patients undergoing bronchoscopy under sedation [2, 3]. Patients with obstructive sleep apnoea (OSA) [4], male sex [5], obesity [5], older age [5] or hypertension [6] are more prone to hypoxemia. Various measures are taken to reduce the incidence of hypoxemia during bronchoscopy under sedation, but the effect is not ideal.

A high-flow nasal cannula (HFNC) can be used to provide an extremely high flow of heated and humidified gas with adjustable temperature and oxygen concentration [7]. A number of studies have evaluated the efficacy of using an HFNC during bronchoscopy [8, 9], as it could prevent the loss of end-expiratory lung volume and improve gas exchange and oxygenation [2]. Some studies have focused on the efficacy of using an HFNC for acute respiratory failure patients [10–12] or lung transplant patients [13] who have an increased risk of hypoxemia, whereas others have investigated patients receiving topical anaesthesia [3] or conscious sedation [14, 15] during bronchoscopy. However, no study has compared the efficacy of nasal cannulas with tight-fitting facemasks in patients at risk of hypoxemia during bronchoscopy, especially under deep sedation. Therefore, this study was conducted to assess whether using an HFNC has distinct advantages of preventing oxygen desaturation in patients at risk of hypoxemia during FB under deep sedation.

## Patients and methods

### Design and study subjects

This study was approved by the local ethics committee (IRB-2021-33). All participants signed a written informed consent form prior to the study. A total of 396 patients were screened, among whom 176 completed the study and had their results analysed at the Cancer Hospital of the University of Chinese Academy of Sciences (Zhejiang Cancer Hospital) from March to April 2021. The study was registered at www.chiCTR.org.cn (ChiCTR2100044105) on 11/03/2021.

Both outpatients and inpatients undergoing FB were recruited for this study. The most frequent indications for bronchoscopy were radiologic changes suggestive of tumours and endobronchial examination before surgery or before radiotherapy in patients with lung tumours. The inclusion criteria included (1) 18 to 80 years of age and (2) at risk of hypoxemia, defined as having a STOP-BANG (snoring, tiredness, observed apnoea, high blood pressure, body mass index [BMI], age, neck circumference, and male sex) [16] score ≥ 3. The exclusion criteria were as follows: (1) American Society of Anaesthesiologists (ASA) class > III; (2) coagulopathy disorders defined by coagulopathy function or a tendency for nose bleeding; (3) severe cardiac disease, including aortic stenosis, mitral stenosis, haemodynamic instability caused by severe arrhythmia, and acute myocardial infarction or cardiac surgery within the last 6 months; (4) severe oxygen desaturation (SpO_2_ < 90% without oxygen supply on admission); (5) upper respiratory tract infection or lung infection; and (6) refusal to participate in this study.

### Study protocol

#### Randomization and blinding

**Fig. 1:**
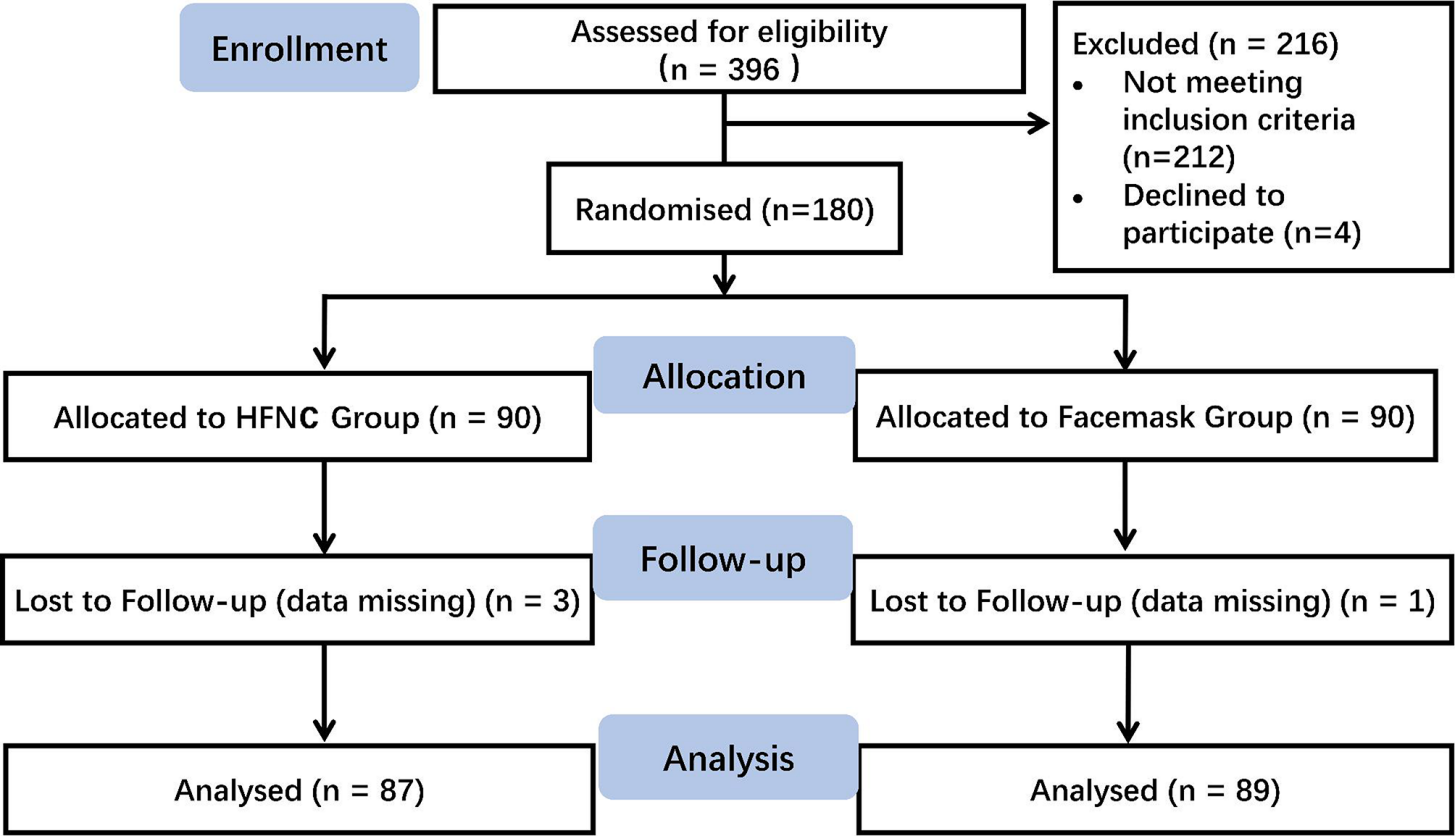
Consolidated Standards of Reporting Trials (CONSORT) flowchart of patient recruitment

The study flowchart is illustrated in Fig. 1. Patients were randomly assigned to the HFNC group or the facemask group in a 1:1 ratio by a random number table. The treatment allocation was placed into a sealed, sequentially numbered and opaque envelope. Investigators were blinded to the study protocol and treatment allocation throughout the study. The investigators recorded the patients’ demographic information, adverse events, real-time oxygen saturation, heart rate and blood pressure on a paper case report form. The anaesthesiologist noticed every change in oxygen saturation and performed the appropriate intervention. The patients, anaesthesiologist, and pulmonologists could not be blinded due to the study design.

### Study intervention

The patients’ demographic information, such as sex, weight, height, smoking status, present illness and history of past illness, was collected. Additionally, the interincisor distance, thyromental distance, and modified Mallampati score (I-IV) were recorded by the investigators. After successful peripheral intravenous access, all patients, before sedation, received 20 min of nebulization with 10 ml of 2% lidocaine via a nebulizer facemask.

**Fig. 2:**
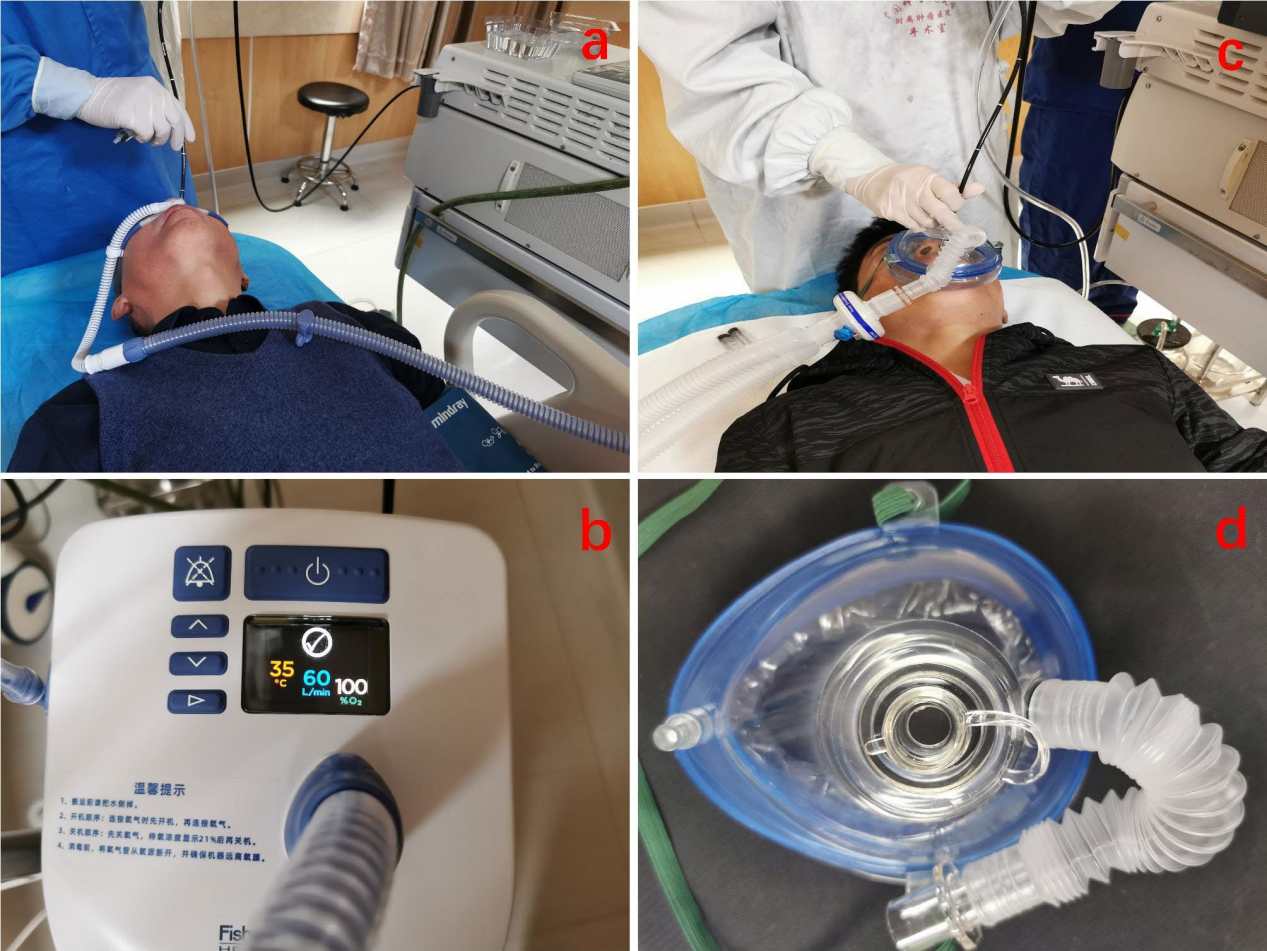
The high-flow nasal cannula and endoscopic facemask. a and b: high-flow nasal cannula device; c and d: endoscopic facemask used in flexible bronchoscopy. Bronchoscopy was performed through the nasal route in a supine position

The baseline oxygen saturation, heart rate, and blood pressure values of the patients were recorded by the investigators. Continuous electrocardiography and pulse oximetry were recorded, and noninvasive blood pressure of the patients was monitored every 5 min throughout the procedure. Patients in the HFNC group received humidified oxygen at a rate of 60 L/min and a concentration of 100% via using an HFNC (AIRVO2, Fisher & Paykel, New Zealand), while those in the facemask group received oxygen at a flow rate of 6 L/min via a tight-fitting facemask (MedPlus Inc., China) attached to a cycle system (Fig. 2). The end-tidal carbon dioxide waveform was monitored to ensure that a tight seal was achieved between the patient and the facemask. Patients in both groups were given supplemental oxygen through the corresponding oxygenation methods for 1 min before sedation. Then, single doses of 0.06–0.1 µg/kg sufentanil and 2–3.5 mg/kg propofol were administered slowly by an anaesthesiologist based on the body weight, age and comorbidities of the patients. The Ramsay sedation score (RSS) was used to assess the level of sedation. Bronchoscopy was performed through the nasal route in a supine position when the RSS was > 4, and 3 ml of 2% lidocaine was sprayed locally over the vocal cords and the trachea. An RSS > 4 was maintained throughout the procedure, and 0.05 mg/kg propofol was given to achieve adequate sedation if necessary. The total dose of propofol used and adverse reactions of patients, such as cough, oppositional behaviour, tachycardia, bradycardia, and hypotension, were recorded. Tachycardia was defined as a heart rate of more than 100 beats per minute or an increase of > 25% from baseline; bradycardia was defined as a heart rate of less than 50 beats per minute or a decrease of > 25% from baseline; and hypotension was defined as a systolic blood pressure less than 90 mmHg or a decrease of > 20% from baseline. Recovery delay was defined as failure to return to baseline clinical status within 2 h.

### Postanaesthesia care unit (PACU)

Patients were transferred to the PACU if their vital signs were stable. All patients received oxygen at 3 L/min through a nasal catheter. After at least 30 min of observation in the PACU, patients were allowed to leave the clinic after their post anaesthetic Aldrete recovery score was assessed. A score of 9 or 10 was required to be discharged. Additionally, airway obstruction and oxygen desaturation in the PACU were recorded by the investigators. Airway obstruction was defined as the tongue falls back against the posterior pharynx, which could be alleviated by the combination of jaw thrust and head-tilt manoeuvres and insertion of an oral/nasal-pharyngeal airway.

### Outcomes and airway interventions

The primary outcome of this study was the incidence of oxygen desaturation in the two groups. The secondary outcomes were airway interventions.

Oxygen desaturation was defined as an SpO_2_ < 90% and was divided into moderate desaturation (75% ≤ SpO_2_ < 90%, lasting < 60 s) and severe desaturation (SpO_2_ < 75% or 75% ≤ SpO_2_ < 90% lasting > 60 s), as recommended by the World Society of Intravenous Anaesthesia (SIVA) International Sedation Task Force [17]. The airway was opened in all patients by the jaw thrust manoeuvre when the SpO_2_ dropped below 95%. When moderate desaturation occurred, treatments including an increase in oxygen flow from 6 L/min to 10 L/min and airway opening by the jaw thrust manoeuvre were provided to the facemask group, while only the latter was given to the HFNC group. For severe desaturation, patients received bag-mask ventilation. If oxygen saturation still did not improve, endotracheal intubation was performed by the anaesthesiologist at his or her own discretion.

### Statistical analysis

The sample size was calculated with PASS version 15.0 (NCSS, LLC, Kaysville, UT, USA). According to the results of our preliminary experiment, the percentages of oxygen desaturation in the HFNC group and facemask group were 5% and 26%, respectively. Herein, we estimated that a sample size of 81 subjects per group would provide 90% power with an alpha of 0.01 using the two independent proportions of Z tests. To compensate for possible dropouts, the sample size was increased to 180 subjects (90 per group).

IBM SPSS Statistics for Windows version 26.0 (IBM Corp., Armonk, NY, USA) was used for the statistical analyses. Categorical variables are presented as numbers (%), and continuous variables are presented as the mean (standard deviation) or median (interquartile range). Normality of continuous variables was assessed by the Kolmogorov–Smirnov test. Continuous variables were analysed with Student’s *t* test or the Mann-Whitney U test, as appropriate. Categorical variables were analysed using the chi-square test or Fisher’s exact test, as appropriate. Bonferroni correction was made for multiple hypothesis tests. The odds ratio (OR) and 95% confidence interval (CI) of the variables possibly associated with the incidence of oxygen desaturation were estimated using multivariate binary logistic regression after adjusting for age, sex, BMI, hypertension, snoring, neck circumference, modified Mallampati score, propofol dose and sufentanil dose. Values of *P* < 0.05 for the 2-tailed test were considered statistically significant.

## Results

### Descriptive data

A total of 396 patients were screened for eligibility, 180 of whom were included in the study. Four patients were excluded because of missing data. The patient characteristics were well balanced between the groups (Table 1).

**Table 1:** The demographic information and medical history of the patients

	HFNC Group (n = 87)	Facemask Group (n = 89)	*P* value
Age (yrs)	64.2 ± 9.3	63.6 ± 7.7	0.661
Sex Male, no. (%) Female, no. (%)	74 (85.1) 13 (14.9)	73 (82.0) 16 (18.0)	0.587
Weight (kg)	64.0 ± 9.9	65. 6 ± 9.1	0.290
BMI (kg/m^2^)	23.6 ± 2.8	23.8 ± 2.9	0.508
ASA physical status ^a^ I/II, no. (%) III, no. (%)	84 (96.6) 3 (3.4)	88 (98.9) 1 (1.1)	0.597
Smoking Status History (pack years)	20 (0 to 40)	24 (0 to 40)	0.759
Current Smoker, no. (%) Past Smoker, no. (%) Never Smoked, no. (%)	20 (23.0) 39 (44.8) 28 (32.2)	30 (33.7) 29 (32.6) 30 (33.7)	0.172
Comorbidity Hypertension, no. (%) Diabetes, no. (%) Heart disease, no. (%) Asthma, no. (%) COPD, no. (%) Lung cancer, no. (%) Oesophagus Cancer, no. (%)	32 (36.8) 5 (5.7) 2 (2.3) 0 (0) 2 (2.3) 17 (19.5) 4 (4.6)	47 (52.8) 2 (2.2) 3 (3.3) 0 (0) 1 (1.1) 15 (16.9) 0 (0)	0.033 0.422 1.000 NS 0.984 0.644 0.123
STOP-Bang Questionnaire Total scores	3 (3 to 4)	3 (3 to 4)	0.282
Snoring, no. (%) Neck circumference > 40 cm, no. (%)	68 (78.2) 12 (13.8)	71 (79.8) 12 (13.5)	0.793 0.952
Modified Mallampati score ^b^, I/II/III/IV	44//34/9/0	46/31/11/1	0.855
Mouth opening ^c^, 1/2/3	0/1/86	0/0/89	0.494
Thyromental Distance ^d^, I/II/III	77/8/2	77/7/5	0.599

Data are presented as numbers (%), means ± standard deviations or medians (interquartile ranges)

Abbreviations: BMI: body mass index; COPD: chronic obstructive pulmonary disease; OSA: obstructive sleep apnoea

^a^ ASA physical status: I: normal healthy patient, II: patient with mild systemic disease that does not limit physical activity, III: patient with severe systemic disease

^b^ Modified Mallampati score: Class I: the entire palatal arch is visible down to the bases of the pillars, Class II: the upper part of the faucial pillars and most of the uvula are visible, Class III: only the soft and hard palates are visible, Class IV: only the hard palate is visible

^c^ Mouth opening:1, one finger; 2, two fingers; 3, three fingers

^d^ Thyromental Distance: I, > 6.5 cm; II, 6-6.5 cm; III, < 6 cm

### Main findings

The facemask group had a higher incidence of oxygen desaturation than the HFNC group (26[29.2%] vs. 4[4.6%], *P* < 0.001) (Table 2). The proportion of moderate and severe desaturation in the facemask group was significantly higher than that in the HFNC group. Therefore, the HFNC group required fewer jaw thrust manoeuvres and bag-mask ventilation than the facemask group. Besides, the patients in the facemask group had more interruptions of bronchoscopy than those in the HFNC group.

**Table 2:** Primary outcome and airway interventions during bronchoscopy

	HFNC Group (n = 87)	Facemask Group (n = 89)	Odds Ratio (95%CI)	*P* value
Primary outcome Nil, no. (%) ^a^	83 (95.4)	63 (70.8)	-	< 0.001 ^b^
Oxygen desaturation, no. (%)	4 (4.6)	26 (29.2)	0.093 (0.028 to 0.313)	
Moderate desaturation, no. (%) severe desaturation, no. (%)	4 (4.6) 0 (0)	18 (20.2) 8 (9.0)	0.163 (0.048 to 0.547) -	0.001 ^b^ 0.005 ^b^
Interventions				
Jaw thrust manoeuvre, no. (%)	5 (5.7)	43 (48.3)	0.041 (0.012 to 0.134)	< 0.001
Increase the flow of oxygen, no. (%)	0 (0)	21 (23.6)	-	< 0.001
Mask ventilation, no. (%)	0 (0)	8 (9.0)	-	0.012
Intubation, no. (%)	0 (0)	0 (0)	-	NS
Number of bronchoscopy interruptions	0 (0)	8 (9.0)	-	0.012

Data are presented as numbers (%)

^a^ Nil was defined as SpO_2_ ≥ 90%.

^b^*P* < 0.0167 was considered statistically significant after Bonferroni correction

No significant difference was detected in adverse reactions, including tachycardia, bradycardia, and hypotension, during bronchoscopy between the two groups (Table 3). There was no difference in the types of diagnostic procedures between the two groups. The propofol and sufentanil dosages and the duration of bronchoscopy between the two groups were not significantly different.

**Table 3:** Adverse events, sedation medications and procedures during bronchoscopy

	HFNC Group (n = 87)	Facemask Group (n = 89)	*P* value
Tachycardia, no. (%)	32 (36.8)	30 (33.7)	0.670
Bradycardia, no. (%)	4 (4.6)	1 (1.1)	0.351
Hypotension, no. (%)	42 (48.3)	37 (41.6)	0.371
Recovery delay, no. (%)	0 (0)	0 (0)	NS
Cardiovascular collapse, no. (%) ^a^	0 (0)	0 (0)	NS
Cardiac arrest, no. (%) ^b^	0 (0)	0 (0)	NS
In PACU Airway obstruction, no. (%) Desaturation, no. (%)	8 (9.2) 0 (0)	15 (16.9) 4 (4.5)	0.132 0.135
Total Propofol dose (mg)	176.8 ± 39.0	172.4 ± 31.9	0.411
Total Propofol dose (mg/kg)	2.8 ± 0.5	2.6 ± 0.4	0.064
Sufentanil dose (µg/kg) Duration of bronchoscopy(s) ^c^	0.07 ± 0.01 300 (214 to 363)	0.07 ± 0.02 300 (180 to 435)	0.323 0.513
Diagnostic procedures Inspection only, no. (%) BAL, no. (%) Bronchial brushing, no. (%) Biopsy, no. (%)	55 (63.2%) 4 (4.6%) 6 (6.9%) 26 (29.9%)	53 (59.6%) 3 (3.4%) 5 (5.6%) 31 (34.8%)	0.872

Data are presented as numbers (%), means ± standard deviations or medians (interquartile ranges)

Abbreviations: PACU: postanesthesia care unit. BAL: bronchoalveolar lavage

^a^ Cardiovascular collapse: clinical evidence of inadequate perfusion

^b^ Cardiac arrest: absence of pulse and loss of heart function

^c^ The duration of bronchoscopy was the duration between the insertion of the bronchoscope and the removal of the bronchoscope

Furthermore, the groups did not differ in the patients’ haemodynamics during bronchoscopy (Table 4). The lowest oxygen saturation of the facemask group was significantly lower than that of the HFNC group. In the PACU, patients in the HFNC group had a higher oxygen saturation than those in the facemask group.

**Table 4:** Haemodynamics before bronchoscopy, after bronchoscopy and in the PACU

	HFNC Group (n = 87)	Facemask Group (n = 89)	*P* value
Before bronchoscopy Mean BP (mmHg) Heart rate (bpm) Oxygen saturation (%) During bronchoscopy Lowest oxygen saturation (%)	107.0 ± 13.5 80.2 ± 16.0 99 (97 to 100) 100 (98 to 100)	111.3 ± 38.7 77.2 ± 15.3 99 (98 to 100) 94 (89 to 100)	0.330 0.206 0.036 < 0.001
After bronchoscopy Mean BP (mmHg) Heart rate (bpm) Oxygen saturation (%)	90.7 ± 15.0 83.2 ± 13.2 100 (99 to 100)	93.7 ± 15.3 80.8 ± 13.8 100 (99 to 100)	0.185 0.236 0.064
In PACU Mean BP (mmHg) Heart rate (bpm) Oxygen saturation (%)	91.0 ± 38.7 74.9 ± 12.6 100 (99 to 100)	88.2 ± 11.7 74.4 ± 12.0 99 (98 to 100)	0.512 0.782 0.005

Data are presented as the means ± standard deviations or medians (interquartile ranges)

Abbreviations: BP: blood pressure; PACU: postanesthesia care unit

## Discussion

This study showed that the incidence of oxygen desaturation and the requirement of airway interventions such as jaw thrust manoeuvres and bag-mask ventilation in patients at risk of hypoxemia were significantly reduced with an HFNC during bronchoscopy under deep sedation.

An HFNC can rapidly wash out CO_2_ in the nasopharyngeal dead space with a high flow of oxygen [18], which can reduce the dead space and generate 3–7 cmH_2_O positive end-expiratory pressure, thereby increasing the end-expiratory lung volume, reopening the alveoli and preventing atelectasis [19]. Additionally, it can reduce the resistance of the upper respiratory tract and reduce respiratory work [20, 21]. Consequently, the use of an HFNC can prevent desaturation in deeply sedated patients undergoing bronchoscopy.

Our results are consistent with previous studies showing that an HFNC may be a useful tool to avoid oxygen desaturation in patients undergoing bronchoscopy under deep sedation. Previous studies have shown that the use of an HFNC is effective for preoxygenation during intubation [22–25]. In addition, using an HFNC significantly decreases the incidence of desaturation during gastroscopy or colonoscopy under sedation [26–30]. As hypoxemia is more likely to develop during bronchoscopy, a number of studies have evaluated the safety and efficacy of using an HFNC during bronchoscopy. However, no study has compared the effect of an HFNC to that of a facemask in deeply sedated patients at risk of hypoxemia undergoing bronchoscopy.

The HFNC method costs approximately ten times as much as the facemask technique; thus, it is impractical to apply it to all patients undergoing bronchoscopy, and it is essential to identify patients at high risk of developing hypoxemia. The STOP-BANG questionnaire is a favoured, straightforward, effective and highly sensitive screening tool to identify patients with OSA [16, 31]. It consists of eight items with yes or no answers related to the clinical features of OSA, such as snoring, male sex, older age, higher BMI, and hypertension. It was reported that when undergoing intravenous anaesthesia, patients with a STOP-BANG score ≥ 3 had a higher incidence of hypoxemia than those with a STOP-BANG score < 3 [32]. Therefore, the STOP-BANG questionnaire was chosen to screen patients at risk of hypoxemia.

Oxygen desaturation occurs frequently during the short length of sedation in patients at risk of hypoxemia undergoing FB. In the present study, the occurrence of desaturation was 29.2% in the facemask group, which was lower than that reported previously [33], which might be related to the higher oxygen flow and the different sedatives used. Additionally, propofol is commonly used as a sedative alone or in combination with opioids during bronchoscopy owing to its properties of rapid onset and smooth recovery [34–37]. As reported, the dose of propofol administered manually was lower than that administered by a continuous infusion pump [37–39]; nevertheless, both sedation regimens had similar good controllability [40]. Due to the short duration of bronchoscopy in our centre, a single dose of propofol administered manually was chosen in this study.

The limitations of this study are as follows: first, to avoid increasing the trauma and economic burden of patients, arterial blood analysis was not performed; thus, the pH, PaO_2_, and PaCO_2_ between the two groups could not be compared. Therefore, whether an HFNC has an effect on the retention of carbon dioxide within a short period is unknown. Second, as the gold standard for diagnosing OSA, polysomnography was not conducted; thus, few data could be provided about the exact number of patients with OSA. Third, the respiratory rate and tidal volume were not monitored, which made it difficult to explain the underlying cause of hypoventilation. The number of apnoea episodes during the procedure was not recorded. Fourth, patients older than 80 years, with upper respiratory tract infections or lung infections were excluded from the present study. Those patients were more prone to develop hypoxemia. Whether an HFNC can prevent the occurrence of desaturation in those patients needs further investigation. In addition, several studies have demonstrated that an increase in the flow rate with an HFNC can yield a higher FiO_2_ [41]. Moreover, a flow rate of > 50 L/min is advisable to obtain the maximal effect of oxygenation. Therefore, an oxygen flow rate of 60 L/min was chosen for the HFNC group; however, such a high flow rate has the potential to cause discomfort for patients and waste oxygen. Therefore, the optimal flow rate in bronchoscopy under deep sedation needs further exploration.

## Conclusion

In conclusion, the use of an HFNC significantly reduced the incidence of oxygen desaturation and the requirement for airway interventions in patients at risk of hypoxemia during bronchoscopy under deep sedation. These results might modify our clinical practice.

## Data Availability

The datasets used and/or analysed during the current study are available from the corresponding author on reasonable request.

## **Abbreviations**

ASA: American Society of Anaesthesiologists
BAL: bronchoalveolar lavage
BMI: body mass index
CI: confidence interval
COPD: chronic obstructive pulmonary disease
HFNC: high-flow nasal cannula
OR: odds ratio
OSA: obstructive sleep apnoea
PACU: postanaesthesia care unit
RSS: Ramsay sedation score
STOP-BANG: snoring, tiredness, observed apnoea, high blood pressure, body mass index, age, neck circumference, and male sex
