# Correction: Incidence of oxygen desaturation using a high-flow nasal cannula versus a facemask during flexible bronchoscopy in patients at risk of hypoxemia: a randomised controlled trial

**DOI:** 10.1186/s12890-022-02252-z

**Published:** 2022-11-28

**Authors:** Wen Zhang, Jiang-Ling Wang, Shuang Fu, Jia-Ming Zhou, Ye-Jing Zhu, Shu-Nv Cai, Jun Fang, Xinzhong Chen, Kangjie Xie

**Affiliations:** 1 Department of Anesthesiology, Research Center for Neuro-Oncology Interaction, The Cancer Hospital of the University of Chinese Academy of Sciences (Zhejiang Cancer Hospital), Institute of Basic Medicine and Cancer (IBMC), Chinese Academy of Sciences, No. 1 Banshan East Road, Gongshu District, Hangzhou, 310022 Zhejiang China; 2 Department of Anaesthesia, Women’s Hospital, Zhejiang University School of Medicine, Xueshi Road #1, Shangcheng District, Hangzhou, 310006 Zhejiang China; 3 Department of Endoscopy, The Cancer Hospital of the University of Chinese Academy of Sciences (Zhejiang Cancer Hospital), Institute of Basic Medicine and Cancer (IBMC), Chinese Academy of Sciences, Hangzhou, Zhejiang China

**Correction: BMC Pulmonary Medicine (2022) 22:389** 10.1186/s12890-022-02188-4

Following publication of the original article, the authors flagged the following errors in the author list, affiliations information, and corresponding author information, respectively: (1) the names of the 8th and 9th authors had been inadvertently duplicated (as Xinzhong Chen and Kangjie Xie, respectively); (2) a 6th and a 7th affiliation were erroneously included, and these affiliations were based on information taken from affiliations 4 and 5, with the result that affiliations 4 and 5 were incomplete (in addition to there being a 6th and a 7th affiliation included in error); (3) the correspondence information of the corresponding authors (on the bottom-left of the article PDF) had been ordered the wrong way around, with Xin-Zhong Chen's information being positioned first instead of that of Kang-Jie Xie.

The published article has now been corrected and the corrected information may be seen in this erratum. The authors thank you for reading and apologize for any inconvenience caused.

